# Emerging Role of Consolidative Radiotherapy After Complete Remission Following R-CHOP Immunochemotherapy in Stage III–IV Diffuse Large B-Cell Lymphoma: A Single Institutional and Case-Matched Control Study

**DOI:** 10.3389/fonc.2021.578865

**Published:** 2021-02-22

**Authors:** Ji Hyun Hong, Han Hee Lee, Seung-Eun Jung, Gyeongsin Park, Joo-Hyun O, Young-Woo Jeon, Byung-Ock Choi, Seok-Goo Cho

**Affiliations:** ^1^ Department of Radiation Oncology, Catholic University Lymphoma Group, Seoul St. Mary’s Hospital, College of Medicine, The Catholic University of Korea, Seoul, South Korea; ^2^ Department of Gastroenterology, Catholic University Lymphoma Group, Seoul St. Mary’s Hospital, College of Medicine, The Catholic University of Korea, Seoul, South Korea; ^3^ Department of Radiology, Catholic University Lymphoma Group, Eunpyeong St. Mary’s Hospital, College of Medicine, The Catholic University of Korea, Seoul, South Korea; ^4^ Department of Pathology, Catholic University Lymphoma Group, Seoul St. Mary’s Hospital, College of Medicine, The Catholic University of Korea, Seoul, South Korea; ^5^ Department of Nuclear Medicine, Catholic University Lymphoma Group, Seoul St. Mary’s Hospital, College of Medicine, The Catholic University of Korea, Seoul, South Korea; ^6^ Department of Hematology, Catholic University Lymphoma Group, Yeouido St. Mary’s Hospital, College of Medicine, The Catholic University of Korea, Seoul, South Korea; ^7^ Department of Hematology, Catholic University Lymphoma Group, Seoul St. Mary’s Hospital, College of Medicine, The Catholic University of Korea, Seoul, South Korea

**Keywords:** consolidation, radiotherapy, advanced-stage, diffuse large B-cell lymphoma, rituximab, complete remission

## Abstract

**Purpose:**

The role of consolidative radiotherapy (RT) after complete-remission (CR) following rituximab combined with cyclophosphamide, doxorubicin, vincristine, and prednisone (R-CHOP) in advanced-stage diffuse large B-cell lymphoma (DLBCL) remains unclear. We retrospectively analyzed the survival outcomes and patterns of failure with our institutional experience.

**Material and Methods:**

Between 2009 and 2018, 206 patients with stage III-IV DLBCL achieved CR after receiving R-CHOP. Propensity-score matching was used to analyze the role of consolidative RT. The consolidative RT group (n = 34) and the R-CHOP alone group (n = 68) were matched at a 1:2 ratio. After propensity-score matching, 102 patients were analyzed.

**Results:**

With a median follow-up of 39.7 months, 26 patients (25.5%) showed local recurrence. Only one patient failed at the previous RT field. RT was delivered to bulky sites, head and neck lesions, testes, and bone with median dose of 30.6 Gy. The most common site of failure was head and neck lesions followed by bulky sites. The 5-year overall survival (OS), progression-free survival (PFS), and isolated-local recurrence free survival (LRFS) were 73.5, 64.0, and 79.9%. In univariate and multivariate analysis, bone marrow involvement and consolidative RT were associated with isolated LRFS (p = 0.006 and 0.032) significantly.

**Conclusion:**

Consolidative RT improved isolated local control. Based on the pattern of failure, we carefully suggest to radiate on initially involved bulky sites or head and neck lesions. Further studies need to be done to find out the optimal radiation dose and selection of RT site.

## Introduction

Diffuse large B-cell lymphoma (DLBCL) is the most common lymphoid neoplasm in adults (1) and the most common non-Hodgkin’s lymphoma (NHL) subtype (2, 3). With heterogeneous pathologic features, it generally has an aggressive clinical course. Approximately 60–70% of patients with DLBCL are initially diagnosed with advanced-stage disease. Although the addition of rituximab, a monoclonal antibody against CD20, to cytotoxic chemotherapy has substantially improved DLBCL survival (4), outcomes remain poor in advanced disease, with a 10-year overall survival (OS) of 43% (5).

In the pre-rituximab era, the role of consolidative radiotherapy (RT) after chemotherapy has been studied in several randomized trials, including the Southwest Oncology Group (SWOG) 8736 trial, the Eastern Cooperative Oncology Group (ECOG) 1484 study, the Groupe d ‘Etudes des Lymphomes de I’Adulte (GELA LNH) 93-1 trial, and the GELA LNH 93-4 trial (6–8). Although these landmark randomized trials aimed to show the potential benefits of RT, consolidative RT did not show significant improvement in survival outcomes. However, in the rituximab era, several single institutional series (9, 10) showed the benefit of consolidative RT. The role of consolidative RT remains unclear but the results of several studies, including the Italian lymphoma study group, Ricover-60 trial, and Min T trial (11, 12) support its beneficial role in early-stage DLBCL, with better local control (LC), progression-free survival (PFS), and OS.

Although consolidative RT is often recommended for early-stage DLBCL, the role of consolidative RT in advanced disease remains unclear (13, 14). Furthermore, patients with stage III or IV DLBCL tend to have treatment failure more often than those with early-stage DLBCL (13). Although several studies have assessed consolidative RT after chemotherapy (15), few studies have evaluated the addition of rituximab for advanced-stage DLBCL (9). This study retrospectively analyzed the survival outcomes and patterns of failure of advanced-stage DLBCL.

## Materials and Methods

### Patients

**Figure 1:**
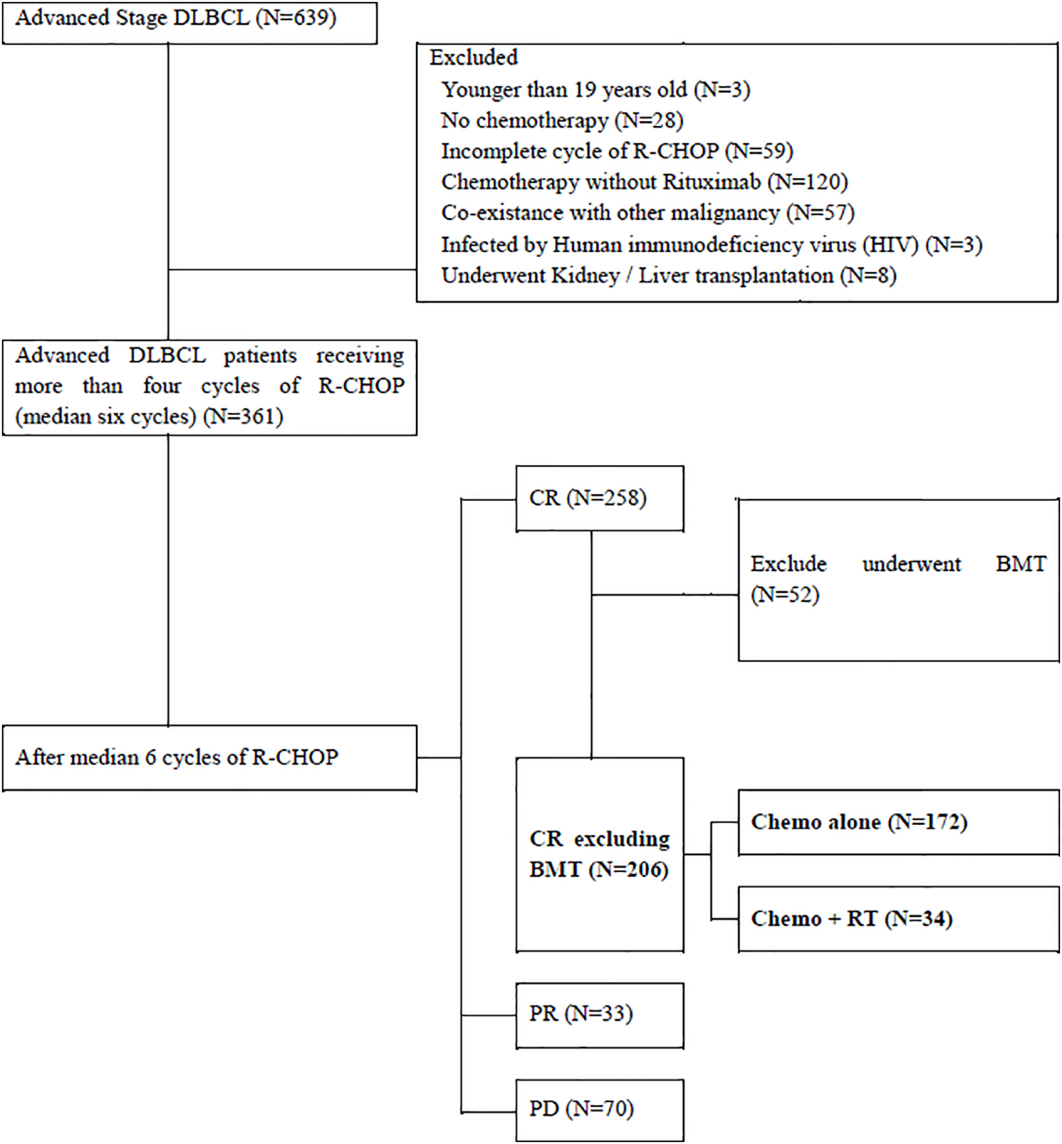
Inclusion Criteria. *DLBCL*, Diffuse Large B-cell lymphoma; *CNS*, Cranial Nervous System; *R-CHOP*, rituximab combined with cyclophosphamide, doxorubicin, vincristine, and prednisone; *CR*, complete remission; *BMT*, bone marrow transplantation; *PR*, partial remission; *PD*, progressive disease; *RT*, radiotherapy.

Between December 2009 and November 2018, 639 patients with histologically proven DLBCL of clinical stage III–IV were reviewed. Patients who were aged <19 years (n = 3); did not receive chemotherapy (n = 28); received fewer than four cycles of rituximab combined with cyclophosphamide, doxorubicin, vincristine, and prednisone (R-CHOP) (n = 59); received chemotherapy without rituximab (n = 120); had other malignancies (n = 57); were under immunosuppressive conditions with human immunodeficiency virus infection (n = 3); and underwent organ transplantations, such as kidney or liver (n = 8) were excluded. Among the 361 patients with stage III–IV DLBCL, only 206 patients who achieved complete response (CR) after receiving more than four cycles of R-CHOP and did not undergo bone marrow transplantation were included. Of these, 172 patients received R-CHOP alone and 34 patients received R-CHOP followed by consolidative RT (**Figure 1**).

Patient charts were reviewed, and the following characteristics were extracted: age, sex, pathologic subtype, Ann Arbor stage, Eastern Cooperative Oncology Group (ECOG) performance status (PS) before treatment, lactate dehydrogenase (LDH) level, extranodal disease involvement, bone marrow involvement, International Prognostic Index (IPI) score, number of R-CHOP cycles, and underlying diseases. In the clinical workup, results of bone marrow biopsy, tissue biopsy, and imaging studies, such as computed tomography (CT) and positron emission tomography (PET)-CT, medical history, physical examination results, and blood test findings were evaluated. All tumors were staged using the Ann Arbor staging system.

### Treatment

The administered chemotherapy regimen was R-CHOP. All patients underwent surveillance studies including PET-CT and CT. Response assessments and outcomes were evaluated according to the response criteria for malignant lymphoma (16). Since the Deauville five-point scale was implemented in 2014, there were few PET scans interpreted without a five-point scale (17). They were reviewed *via* medical charts from the Catholic University Lymphoma Group. CR was defined as the disappearance of all diseases on CT or Deauville score 1 to 3 after R-CHOP (17, 18).

Based on decisions from a multidisciplinary team, the Catholic University Lymphoma Group, including radiation and medical oncologists, radiologists, pathologists, and nuclear radiologists, 34 patients were administered consolidative RT as part of the initial therapy.

### Propensity-Score Matching and Statistical Analysis

To reduce selection bias and potential confounding effects of treatment, propensity-score matching with 1:2 matching was performed. The covariates selected for matching were pathologic subtype, Ann Arbor stage, bone marrow involvement, International Prognostic Index (IPI) score, number of R-CHOP cycles, LDH levels, and underlying diseases. Propensity-score matching was performed using “nearest-neighbor matching” without replacement. A total of 34 patients in the R-CHOP followed by consolidative RT group, and 68 patients in the R-CHOP alone group were matched at a 1:2 ratio. After matching, statistical survival rates and failure patterns were analyzed.

Descriptive statistics were used to identify clinical characteristics between the two groups, with and without consolidative RT. Non-continuous values were compared using the Mann Whitney U-test, and continuous variables were presented as medians and compared using the t-tests.

The actuarial 5-year survival rates were calculated. OS was defined as the time from diagnosis until death as a result of any cause or the last follow-up date. PFS was defined as the time from diagnosis until disease progression or death. Local recurrence (LR) was defined as failure at the initial sites with a Deauville score of 4–5, and distant recurrence (DR) as failure outside the initial sites. Local recurrence free survival (LRFS) and distant recurrence free survival (DRFS) were defined as the time from completion of chemotherapy until local or distant recurrence. Furthermore, in-field failure was defined as recurrence within the previous RT field. Since not every initially involved site was included in the RT field, there is out-field LR, which is recurrence in initially involved sites but outside the RT field. Survival functions were estimated using the Kaplan-Meier method and compared by log-rank tests for univariate analysis. The Cox proportional hazards model was used in the multivariate analysis. All tests were two-sided, and p-values <0.05 indicated statistical significance. All statistical analyses were performed using IBM SPSS Statistics for Windows, version 23.0 (IBM Corp., Armonk, NY, USA) and R version 3.6.3 (R Development Core Team, Vienna, Austria).

## Results

### Patient Characteristics

A total of 102 patients after propensity-score matching were analyzed. Patient characteristics are summarized in **Table 1**. The median age at diagnosis was 57.5 years (range, 19.0–81.0). There were 56 men (54.9%) and 46 women (45.1%). According to the Ann Arbor staging system, 19 (18.6%) and 83 (81.4%) patients had stage III and stage IV diseases, respectively.

**Table 1:** Patients’ characteristics.

Characteristics	All (n=102)	R-CHOP alone (n=68)	R-CHOP + RT (n=34)	P value
Age at diagnosis				0.989
Median	57.5	57	58	
Range	19.0–81.0	21.0–81.0	19.0–79.0	
Gender				1.000
Male	56 (54.9%)	37 (54.4%)	19 (55.9%)	
Female	46 (45.1%)	31 (45.6%)	15 (44.1%)	
Pathologic subtype				0.859
ABC	63 (61.8%)	43 (63.2%)	20 (58.8%)	
GCB	27 (26.5%)	18 (26.5%)	9 (26.5%)	
T cell rich	1 (1.0%)	1 (1.5%)	0 (0.0%)	
NOS	11 (10.8%)	6 (8.8%)	5 (14.7%)	
Ann arbor stage				1.000
Stage III	19 (18.6%)	13 (19.1%)	6 (17.6%)	
Stage IV	83 (81.4%)	55 (80.9%)	28 (82.4%)	
Performance status (ECOG-PS)				1.000
0–1	71 (69.6%)	47 (69.1%)	24 (70.6%)	
2–4	32 (31.4%)	21 (30.9%)	10 (29.4%)	
LDH level				**0.015**
Normal (250–450 IU/L)	28 (27.5%)	15 (22.1%)	13 (38.2%)	
Elevated	74 (72.5%)	53 (77.9%)	21 (61.8%)	
Extranodal disease involvement				0.935
<2	25 (24.5%)	16 (23.5%)	9 (26.5%)	
≥2	77 (75.5%)	52 (76.5%)	25 (73.5%)	
Bone marrow involvement				0.688
No	76 (74.5%)	52 (76.5%)	24 (70.6)	
Yes	26 (25.5%)	16 (23.5%)	10 (29.4%)	
IPI score				0.327
Low 0–1	6 (5.9%)	4 (5.9%)	2 (5.9%)	
Low-intermediate 2	21 (20.6%)	11 (16.2%)	10 (29.4%)	
High-intermediate 3	30 (29.4%)	20 (29.4%)	10 (29.4%)	
High 4–5	45 (44.1%)	33 (48.5%)	12 (35.3%)	
Number of R-CHOP cycles				1.000
6 cycle	58 (56.9%)	39 (57.4%)	19 (55.9%)	
7 cycle	5 (4.9%)	3 (4.4%)	2 (5.9%)	
8 cycle	39 (38.2%)	26 (38.2%)	13 (38.2%)	
Follow-up				0.699
Median	39.7	39.9	39.2	
Range	6.8–125.1	6.8–119.2	9.6–125.1	

ABC, activated B-cell; GCB, germinal center B-cell; NOS, Not otherwise specified; ECOG PS, Eastern Cooperative Oncology Group Performance Status; LDH, lactate dehydrogenase; IPI, International Prognostic Index; R-CHOP, rituximab combined with cyclophosphamide, doxorubicin, vincristine, and prednisone; RT, radiotherapy.

In bold, p value with less than 0.05.

A comparison of characteristics between patients who received R-CHOP alone and those who received consolidative RT after achieving CR is also shown in **Table 1**. A significant difference in LDH level (p = 0.015) was observed between the two groups. Characteristics such as patient age (p = 0.989), gender (p = 1.000), pathologic subtype (p = 0.859), Ann arbor stage (p = 1.000), performance status (p = 1.000), extranodal disease involvement (p = 0.935), bone marrow (BM) involvement (p = 0.688), IPI score (p = 0.327), and number of R-CHOP cycles (p = 1.000) did not significantly differ between groups.

All 102 patients received more than six cycles (range, 6–8) of R-CHOP. After receiving immunochemotherapy, each patient was evaluated by PET-CT. Details on RT are described in **Table 2**. RT was administered at a median of 5.0 weeks (range, 2.6–13.1) after completion of R-CHOP. A total of 32 patients (94.1%) started RT within 8 weeks after completion of R-CHOP, except two patients who had to recover from previous treatment. The median dose of consolidative RT was 30.6 Gy (range, 30.0–50.4 Gy), and the median fraction size was 180 cGy (range, 180–300 cGy). Moreover, 27 patients (79.4%) received ≤30.6Gy. RT was administered to initially bulky sites (≥5 cm), head and neck lesions, testes, and bony lesions using 3D RT (n = 23, 67.6%) or intensity-modulated RT (n = 11, 32.4%). No patients received RT at all involved sites. Involved-field radiotherapy (IFRT) was administered to 18 (52.9%) patients, and involved-site radiotherapy (ISRT), which delivers radiation only to the initially involved sites, to 16 (47.1%) patients.

**Table 2:** Characteristics of Radiation Therapy.

RT character	Number of Patients (%)
Timing of RT (interval of RT start date and R-CHOP end date)	
≤6 week	25 (73.5%)
>6 week	9 (26.5%)
Radiation Dose, Gy	
≤30.6	27 (79.4%)
>30.6	7 (20.6%)
RT technique	
3DRT	23 (67.6%)
IMRT	11 (32.4%)
RT field	
ISRT	16 (47.1%)
IFRT	18 (52.9%)
RT duration	
≤4 weeks	28 (82.4%)
>4 weeks	6 (17.6%)
RT sites	
Bony sites	6 (17.6%)
Bulky sites (≥5 cm)	17 (20.6%)
Head and neck lesions	8 (23.5%)
Testes	3 (8.8%)

RT, radiotherapy; R-CHOP, rituximab combined with cyclophosphamide, doxorubicin, vincristine, and prednisone; 3DRT, 3-demensional radiotherapy; IMRT, intensity-modulated radiotherapy; ISRT, involved-site radiotherapy; IFRT, involved-field radiotherapy.

### Patterns of Failure

With a median follow-up of 39.7 months (range, 6.8–125.1), 33 patients (32.4%) showed recurrence (**Table 3**). LR occurred in 26 patients (25.5%) with and without DR, and DR alone occurred in 7 patients (6.9%). Ten patients (9.8%) showed both LR and DR.

**Table 3:** Patterns of failure.

	All (n = 102)		R-CHOP alone (n = 68)		R-CHOP+RT (n = 34)		P value
	n	%	n	%	n	%	
Any recurrence	33	32.4	25	36.8	8	23.5	0.180
LR only	16	15.7	14	20.6	2	5.9	0.055
DR only	7	6.9	5	7.4	2	5.9	0.783
Both LR and DR	10	9.8	6	8.8	4	11.8	0.639

LR, local recurrence; DR, distant recurrence; R-CHOP, rituximab combined with cyclophosphamide, doxorubicin, vincristine, and prednisone; RT, radiotherapy.

Of 68 patients who received R-CHOP alone, 14 (20.6%) showed isolated LR, 5 (7.4%) showed isolated DR, and 6 (8.8%) showed both LR and DR. Isolated LR was defined as LR without DR, and isolated DR was defined as DR without LR. Twenty patients showed LRs. The most common site of LR was head and neck lesions, which was observed in 10 patients (50.0%). Of the 14 patients with progression to isolated LR, 6 (42.9%) developed LR in head and neck lesions. The second most common site of LR was lymph nodes with initially bulky sizes (>5 cm) which was observed in 7 patients (35%).

Of 34 patients who received R-CHOP with consolidative RT, 2 (5.9%) showed isolated LR, 2 (5.9%) showed isolated DR, and 4 (11.8%) showed both LR and DR. Although the difference was marginally significant, only two patients who received consolidative RT showed isolated LR (5.9%). Furthermore, in-field failure after consolidative RT occurred in only one patient, which suggested that local control was related to consolidative RT.

### Survival Outcomes

**Figure 2:**
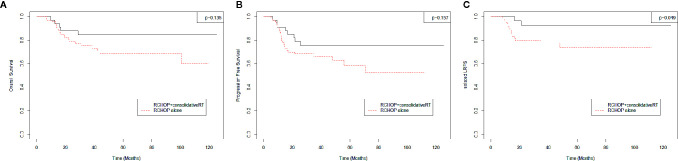
**(A)** Overall survival, **(B)** Progression Free Survival, **(C)** Isolated-Local Recurrence Free Survival.

The estimated actuarial 5-year OS, PFS, LRFS, DRFS, and isolated-LRFS rates were 73.5%, 64.0%, 68.4%, 80.1%, and 79.9%, respectively. Consolidative RT significantly improved the 5-year isolated-LRFS (73.6 *vs.* 92.9%, p = 0.049) compared to R-CHOP alone (**Figure 2C**). However, consolidative RT did not show significant improvement in OS (**Figure 2A**) or PFS (**Figure 2B**).

Univariate analysis (**Table 4**) showed that elevated LDH level (p = 0.015), extranodal disease involvement (p = 0.040), and high intermediate to high IPI score (p = 0.009) significantly decreased the 5-year OS. Elevated LDH levels were also associated with significantly decreased 5-year PFS (p = 0.042). Patients with Ann Arbor stage IV and elevated LDH levels showed significantly decreased 5-year DRFS (p = 0.046 and p = 0.028), while age, gender, pathologic subtype, stage, LDH level, extranodal disease involvement, bone marrow involvement, IPI score, and consolidative RT did not show any significance for LRFS. In the multivariate analyses, IPI score was a significant factor for OS (hazard ratio, 9.033, p = 0.031), and LDH level was a significant factor for PFS (hazard ratio, 3.175, p = 0.019).

**Table 4:** Univariate and Multivariate Analysis for Survival Outcomes.

Characteristics	Overall Survival				Progression Free Survival			
	5yr OS (%)	Univariate (p)	Hazard Ratio (95% CI)	Multivariate(p)	5yr PFS (%)	Univariate (p)	Hazard Ratio (95% CI)	Multivariate(p)
Age at diagnosis		0.109		0.490		0.664		0.699
≤60	83.1		Referent		67.1		Referent	
>60	62.0		1.361 (0.568–3.263)		60.1		1.173 (0.523–2.633)	
Gender		0.534		0.391		0.983		0.394
Female	73.7		Referent		55.5		Referent	
Male	72.5		1.498 (0.595–3.769)		67.8		1.403 (0.643–3.061)	
Pathologic subtype		0.084		0.068		0.663		0.582
Non-GCB	68.3		Referent		62.4		Referent	
GCB	88.6		0.298 (0.081–1.092)		69.0		0.788 (0.336–1.844)	
Ann arbor stage		0.172		0.579		0.129		0.261
Stage III	89.2		Referent		57.9		Referent	
Stage IV	70.4		1.557 (0.326–7.434)		62.2		2.091 (0.577–7.576)	
Performance status (ECOG-PS)		0.691		0.400		0.067		0.025
0–1	75.4		Referent		57.3		Referent	
2–4	69.8		0.683 (0.281–1.660)		77.8		0.359 (0.146–0.881)	
LDH level		**0.015**		0.107		**0.042**		**0.019**
Normal (250–450 IU/L)	92.0		Referent		80.5		Referent	
Elevated	66.7		4.012 (0.741–21.705)		58.3		3.175 (1.214–8.305)	
Extranodal disease involvement		**0.040**		0.218		0.786		0.820
<2	91.5		Referent		54.2		Referent	
≥2	68.1		2.984 (0.523–17.020)		68.1		1.136 (0.380–3.396)	
Bone marrow involvement		0.952		0.647		0.173		0.154
No	77.0		Referent		66.6		Referent	
Yes	65.3		1.262 (0.465–3.423)		57.4		1.751 (0.811–3.782)	
IPI score		**0.009**		**0.031**		0.400		0.846
Low to low intermediate	96.0		Referent		60.4		Referent	
High intermediate to high	66.2		9.033 (1.221–66.800)		64.1		0.860 (0.187–3.946)	
Number of R-CHOP cycles		0.677		0.642		0.682		0.312
6	70.0		Referent		57.8		Referent	
7–8	78.5		0.807 (0.326–1.995)		67.6		0.678 (0.320–1.439)	
Consolidative RT		0.135		0.450		0.157		0.237
No	68.6		Referent		58.2		Referent	
Yes	84.7		0.676 (0.244–1.870)		75.1		0.608 (0.266–1.387)	

GCB, germinal center B-cell; ECOG PS, Eastern Cooperative Oncology Group Performance Status; LDH, lactate dehydrogenase; IPI, International Prognostic Index; R-CHOP, rituximab combined with cyclophosphamide, doxorubicin, vincristine, and prednisone; RT, radiotherapy; OS, overall survival; CI, confidence interval; PFS, progression free survival.

In bold, p value with less than 0.05.

In the univariate analysis of isolated-LRFS (**Table 5**), bone marrow involvement and consolidative RT showed significance (p = 0.013 and p = 0.049, respectively). The absence of bone marrow involvement and presence of consolidative RT also improved isolated-LRFS in the multivariate analyses (hazard ratio, 3.973, p = 0.006, and hazard ratio, 0.195, p = 0.032, respectively).

**Table 5:** Univariate and Multivariate Analysis for Isolated-LRFS.

Characteristics	Isolated-Local Recurrence Free Survival.			
	5-yr isolated-LRFS (%)	Univariate (p)	Hazard Ratio (95% CI)	Multivariate(p)
Age at diagnosis		0.146		0.242
≤60	86.9		Referent	
>60	70.9		2.161 (0.594–7.861)	
Gender		0.468		0.338
Female	78.3		Referent	
Male	79.8		1.853 (0.525–6.537)	
Pathologic subtype		0.726		0.794
Non-GCB	80.1		Referent	
GCB	80.7		1.195 (0.313–4.568)	
Ann arbor stage		0.874		0.518
Stage III	57.9		Referent	
Stage IV	81.6		0.595 (0.123–2.880)	
Performance status (ECOG-PS)		0.081		0.068
0–1	76.8		Referent	
2–4	89.4		0.195 (0.044–0.866)	
LDH level		0.505		0.628
Normal (250–450 IU/L)	83.5		Referent	
Elevated	78.8		1.445 (0.326–6.409)	
Extranodal disease involvement		0.879		0.659
<2	75.9		Referent	
≥2	81.7		0.682 (0.124–3.739)	
Bone marrow involvement		**0.013**		**0.006**
No	85.0		Referent	
Yes	65.7		3.973 (1.476–10.693)	
IPI score		0.366		0.578
Low to low intermediate	86.0		Referent	
High intermediate to high	77.6		1.860 (0.209–16.531)	
Number of R-CHOP cycles		0.958		0.534
6	81.3		Referent	
7–8	79.4		0.684 (0.206–2.266)	
Consolidative RT		**0.049**		**0.032**
No	73.6		Referent	
Yes	92.9		0.195 (0.044–0.866)	

GCB, germinal center B-cell; ECOG PS, Eastern Cooperative Oncology Group Performance Status; LDH, lactate dehydrogenase; IPI, International Prognostic Index; R-CHOP, rituximab combined with cyclophosphamide, doxorubicin, vincristine, and prednisone; RT, radiotherapy; CI, confidence interval; LRFS, local recurrence free survival

In bold, p value with less than 0.05.

Additionally, an analysis based on pathology features was performed. Five patients showed c-MYC protein expression, and all of them showed either BCL 2 or BCL 6 protein expression, while one patient showed c-MYC, BCL 2, and BCL 6 protein expression. Moreover, four patients showed EBV-related DLBCL. Patients with c-MYC and BCL 2 protein expression showed significantly worse DRFS (p = 0.011 and p = 0.026, respectively). EBV-related DLBCL significantly decreased OS (p = 0.011), PFS (p = 0.011), LRFS (p = 0.004), and isolated LRFS (0 = 0.006). However, the number of patients was small, and the statistics should be carefully interpreted with a small number of cases.

In the consolidative RT group, total dose (≤30.6 *vs.* >30.6 Gy), fraction size (≤180 *vs.* >180 cGy), and RT timing (≤6 weeks *vs.* >6 weeks) were not significant factors for OS (p = 0.997, p = 0.237, and p = 0.836, respectively) or PFS (p = 0.758, p = 0.241, and p = 0.387, respectively).

## Discussion

The role of consolidative RT in advanced-stage DLBCL after R-CHOP remains controversial. There is not any randomized controlled trial comparing treatment outcomes between R-CHOP and R-CHOP followed by consolidative RT for complete responders with advanced-stage DLBCL. However, in the GELA LNH 98-5 trial, 24% of patients who achieved CR after R-CHOP showed relapses; among them, 80% had stage III and IV DLBCL. In the era of rituximab, the 5-year survival rate for advanced-stage DLBCL is still approximately 60%, with a disease relapse rate of 50%. Consolidative treatment to reduce relapse and improve survival is needed. Some studies have shown excellent LC after consolidative RT, especially for patients with initially bulky diseases (9, 13).

This study evaluated our experience in administering consolidative RT for advanced-stage DLBCL. We compared the survival outcomes and analyzed failure patterns of patients with R-CHOP alone and R-CHOP followed by consolidative RT. Our analyzed treatment outcomes showed that 26 patients (25.5%) failed at the initially involved sites even after achieving CR following R-CHOP with or without consolidative RT. Among them, 12 (46.2%) and 11 patients (42.3%) had LR at initially bulky lymph nodes >5 cm in size and head and neck lesions, respectively.

In this study, OS did not significantly differ between the R-CHOP alone and R-CHOP followed by the consolidative RT group (p = 0.135, 5-year OS R-CHOP alone *vs.* R-CHOP+RT 68.6 *vs.* 84.7%). The same tendency was observed for PFS (p = 0.175, 5-year PFS R-CHOP alone *vs.* R-CHOP+RT 58.2 *vs.* 75.1%). Although there was no statistical difference in PFS, there was a trend that PFS was better in the consolidation RT group. Data from the MDACC (9), Duke (10), and Emory (13) showed that consolidative RT after achieving CR from R-CHOP improved OS, PFS, and LRFS in advanced-stage DLBCL. The MDACC (9) study included all stages, and only 14.0% of patients with advanced-stage DLBCL received consolidative RT. In contrast, the Duke (10) and Emory (13) studies included only patients with stage III–IV. Moreover, 48.1% and 12.7% of patients with advanced-stage DLBCL received RT, respectively. Similar to the MDACC (9) and Emory (13) studies, 16.5% of patients with advanced-stage DLBCL were treated with consolidative RT in this study. In all patients, survival outcomes were relatively comparable with those of other studies. However, for only the consolidative RT group, the 5-year PFS (75.1%) and LRFS (80.1%) were inferior to those of the Duke (10) (82.0 and 92.0%, respectively) and Emory (13) (85.1 and 91.7%, respectively) studies. Unlike the Duke (10) and Emory (13), which included 27.8 and 42.0% of patients with stage III and 72.2 and 58% of patients with stage IV, respectively, this study included 18.6% of patients with stage III and 81.4% of patients with stage IV. With a greater proportion of patients with stage IV, 10 patients (38.5%) with local failure had distant failure. Of note, 5-year isolated-LRFS, which did not include DR, showed better outcomes (in all patients, R-CHOP alone, and consolidative RT group, 79.9, 73.6, and 92.9%, respectively).

In the consolidative RT group, six patients (17.6%) developed LR with four patients showing LR with DR. Furthermore, only one patient showed in-field failure, who also showed DR at the same time. However, in the R-CHOP alone group, 20 patients (29.4%) showed LR. In both the R-CHOP alone group and R-CHOP followed by consolidative RT group, more patients showed LR (29.4 and 17.6%, respectively) than isolated-DR (7.4 and 5.9%, respectively). Seventeen patients showed DR including 9 patients who were initially diagnosed with stage IV disease.

Although rituximab improved the survival outcomes of DLBCL, LR was the dominant cause of failure. Particularly, patients who initially had bulky lesions or head and neck lesions need to be aware of LR. In the era of intensity-modulated radiation therapy (IMRT), RT for head and neck lesions became more feasible with less toxicity. Kawk et al. (19) also reported excellent LC of consolidative RT in DLBCL of head and neck lesions. Several studies have also reported that bulky disease is an important prognostic factor for local failure (4, 9, 11, 12, 14, 20). Even though this study did not show statistical significance, the failure pattern indicated the tendency of local failure with bulky disease >5 cm. Some studies have shown that a bulky tumor burden results in a lack of vascular flow that impairs drug delivery (21). With this explanation, the advent of rituximab would lessen this effect. Even though it is difficult to administer RT to all initially involved sites because of concerns on toxicity, the frequency of failure in this study suggests the application of consolidative RT, especially to the initially involved bulky sites and head and neck lesions.

This study has several limitations. First, as a retrospective study, there was selection bias between the two groups. The number of patients between the two groups was imbalanced. There is no consensus regarding the indications for referral of patients with consolidative RT. Usually, in our institution, patients with worse prognostic factors with ABC (activated B-cell) pathologic subtypes, bone involvement, and initially bulky sized lesions tend to receive consolidative RT. However, with propensity-score matching analysis, there was no significant difference in the characteristics of patients. Moreover, patients received combined modality treatment, which made it difficult to compare identical conditions. Second, the follow-up period of 39.7 months is insufficient, which may have affected the accuracy of the statistical analyses. Third, 26.5% of patients showed >6 weeks of interval between the end of chemotherapy and start of RT. These were longer the typical range for consolidative RT, which is 4 to 6 weeks. Finally, the relatively small number of patients might also affect the accuracy of the statistics.

Based on the results of this study and previous studies, consolidative RT is beneficial for local control in advanced-stage DLBCL and is a promising treatment option. Especially, there is only one in-field failure after consolidative RT in our study, which supports the outstanding local control rate of RT. Univariate and multivariate analyses showed that consolidative RT improved 5-year isolated-LRFS (p = 0.049 and p = 0.032, respectively), while bone marrow involvement statistically significantly decreased 5-year isolated-LRFS (p = 0.013 and p = 0.006, respectively). Consolidative RT can be considered for improvement in local control, even though it is difficult to insist administrating RT in all patients strongly, since this study is a case-matched control study. Also, it is hard to insist that RT would be more helpful in patients with bone marrow involvement as there is no statistically definitive relationship between RT and bone marrow involvement. However, since consolidative RT showed improved isolated-LRFS, applying consolidative RT might be considered in bone marrow involved patients with worse isolated-LRFS as further local treatment. As the pattern of failure showed, we carefully suggest to radiate on initially involved bulky sites or head and neck lesions. However, further studies on the optimal radiation field and dose evaluation are necessary. Further prospective studies with larger sample sizes are required to validate the role of radiation in advanced-stage DLBCL.

## Data Availability Statement

Datasets are available on request. The raw data supporting the conclusions of this article will be made available by the authors, without undue reservation.

## Ethics Statement

This study was reviewed and approved by the institutional review board (IRB) of Seoul St. Mary’s Hospital. (IRB number: KC19RESI0705) Because of the retrospective nature of the study, patient consent for inclusion was waived.

## Author Contributions

Design of the study: SGC, and BOC. Collection of the data: HHL, SEJ, KSP, YWJ and HJH. Analysis and interpretation of the data: BOC and HJH. Writing and drafting of the manuscript: HJH. Revision of the manuscript: All authors contributed to the article and approved the submitted version.

## Conflict of Interest

The authors declare that the research was conducted in the absence of any commercial or financial relationships that could be construed as a potential conflict of interest.
